# Impact of exposure to community and school violence during adolescence in the African context: systematic review

**DOI:** 10.1192/bji.2025.10043

**Published:** 2025-11

**Authors:** Marinos Bomikazi Lupindo, Hjördis Lorenz, Sam French, Paul Salkovskis

**Affiliations:** 1 DPhil candidate, Department of Experimental Psychology, University of Oxford, Oxford, UK; 2 Research clinical psychologist, Department of Experimental Psychology, University of Oxford, Oxford, UK; 3 Clinical psychologist, Oxford Institute of Clinical Psychology Training, University of Oxford, Warneford Hospital, Oxford, UK; 4 Professor, Experimental Psychology Department, University of Oxford, Oxford, UK.

**Keywords:** Community, school, violence, adolescents, mental health

## Abstract

**Background:**

Child and adolescent exposure to community and school violence in Africa is pervasive, with significant longer-term consequences for mental health and life outcomes.

**Aims:**

To synthesise research on the impact of exposure to community and school violence, in terms of mental health and adjustment outcomes. The review focuses on adolescents in countries on the African continent, summarising existing knowledge regarding the impact on mental health and adjustment outcomes of different types of violence, and the associated mediating and/or moderating factors.

**Method:**

We used the Preferred Reporting Items for Systematic Review and Meta-Analysis protocols (PRISMA-P) to conduct a systematic narrative review (PROSPERO registration CRD42023390724). PsycInfo, MEDLINE, Global Health and Web of Science databases were searched and 36 articles were included in the review. These studies were conducted in countries within Africa among adolescents (10–19 years of age) exposed to violence in their schools and/or communities, and investigated mental health and adjustment outcomes related to violence exposure.

**Results:**

Adolescents exposed to violence in their schools and communities have increased risk of negative outcomes in areas of psychological, social, behavioural and academic functioning that persist over time. Several mediating and/or moderating variables, such as social support, school climate and negative appraisals, were found.

**Conclusions:**

Exposure to violence in school and the community has a significant and lasting impact on mental health and adjustment which can be exacerbated and/or ameliorated by several mediating and moderating factors. Future research will benefit from the development and evaluation of interventions that deploy early identification and of secondary prevention interventions which could mitigate effects of exposure to violence for youth in high-risk contexts and emerging economies that face additional economic challenges.

Children and adolescents spend significant time in their school and community environments, with adolescents’ increased sense of independence increasing their unsupervised circulation. These environments play a significant role in learning and development, making them critical for safeguarding physical and psychological well-being.^1^ Children and adolescents in contexts such as those found in African countries experience high levels of community and school violence (at least one victimisation experience: 40–98%; poly-victimisation: 38.1–93%), and this increased risk of exposure to violence may increase the risk of negative outcomes among young people in these settings.^2^^–^^9^ In contrast, North American youth living in urban areas with higher rates of violence start at lower rates of exposure (17–23%)^10^ than those in countries in Africa. Furthermore, the rate of poly-victimisation (12–33%) in high-income countries (HICs)^10,11^ is lower than in countries in Africa. The high prevalence rates and proximal occurrence of community and school violence among adolescents in regions such as those found in African countries suggests an urgent need for violence prevention measures and secondary prevention interventions to prevent longer-term effects.

## Long-term consequences of violence exposure

Increasing research points to detrimental longer-term consequences of exposure to community and school violence for the mental health and life outcomes of children and adolescents.^12,13^ Exposure to violence and trauma, especially during early childhood, has a significant impact on psychological, cognitive, social, behavioural, occupational and physical areas of functioning.^14–16^ These challenges continue into young adulthood, further affecting individuals’ life outcomes^17,18^ and increasing risk for trauma reenactments (revictimisation and/or perpetration of violence).^19,20^ This progressive and cyclic effect of violence among children and adolescents in Africa draws attention to the need to further understand the factors related to this exposure to violence and its impact. This understanding can help early identification of vulnerable youth and development of interventions in contexts of continued violence.

### Challenges faced by African countries

Although the African continent has the youngest population globally (70% of the population is under 30 and it is estimated that by 2055, Africa’s child population will reach 1 billion),^21,22^ research on the impact of childhood exposure to violence and trauma in this context has only recently grown.^16,23^ However, there is limited research on modifiable factors that mediate and/or moderate the effects of various forms of community violence (including poly-victimisation) on adolescents’ development and mental health.^23–27^ Increased understanding of these factors may inform focused interventions for vulnerable adolescents in high-violence contexts such as those in African countries. Du Plessis et al^26^ and Schwartz et al^28^ emphasised this as being crucial, especially given that low-and-middle-income countries (LMICs) are disadvantaged by limited resources, with mental health interventions being the most poorly resourced,^29,30^ increasing the vulnerabilities faced by adolescents in these settings. Therefore, the goal should be to predict and improve mental health and adjustment problems that have an impact on life outcomes of adolescents growing up exposed to violence and trauma in their communities.

### Current research

This systematic review seeks to set the scene for further research by synthesising knowledge about the relationship between being exposed to community/school violence during adolescence and poor mental health and adjustment outcomes among adolescents in African countries. It also seeks to investigate whether there is association between different categories of violence and abuse and varied negative outcomes among these adolescents. Finally, the review seeks to identify factors that are associated with mediating and/or moderating the effects of community/school violence exposure on mental health and adjustment outcomes among adolescents in Africa.

## Method

### Definitions

In this review, the terms ‘Africa’ or ‘African continent’ encompass countries situated to the south of the Sahara Desert, i.e. sub-Saharan Africa. ‘Community violence’ refers to violence that occurs in neighbourhoods and streets, and ‘school violence’ refers to violence occurring within schools. This includes but is not limited to physical, sexual and interpersonal forms (both direct and indirect). ‘Victimisation’ refers to direct forms of violence, and ‘witnessing’ refers to indirect forms of violence. ‘Abuse’ refers to any form of abuse, including physical, sexual, emotional, psychological and socio-economic abuse, as well as neglect, maltreatment and bullying. ‘Adjustment problems’ refers to behavioural, physical, cognitive and psychosocial symptoms.

### Study design

The systematic narrative review design was employed and included both qualitative and quantitative studies (including prospective, comparative and correlational longitudinal studies, as well as descriptive and categorical cross-sectional studies).

### Inclusion and exclusion criteria

#### Population

Studies were included if they had samples who were adolescents, i.e. aged 10–19 years as classified by the World Health Organization.^31^

#### Exposure type

Our interest was in violence or trauma experienced in the community or school context in Africa (see above definitions). Participants could be victims, witnesses or both. Only original studies that collected primary data using questionnaires, interviews or both were included (details on measures used can be found in Supplementary Tables B1 and B2, available online at https://doi.org/10.1192/bji.2025.10043). Studies were excluded if they were solely focused on specific groups, such as refugees, war victims, young people in juvenile detention centre and/or systems, HIV populations or on violence witnessed on the television. Systematic reviews, meta-analyses, case studies and studies on domestic violence, dating or inter-partner violence, war or political violence were also excluded.

#### Outcomes of interest

Studies reporting the impact that the violence, abuse or maltreatment had on their sample were included. Studies that reported mediating and/or moderating factors of violence exposure were also included.

### Search strategy

The Preferred Reporting Items for Systematic Reviews and Meta-Analysis Protocols (PRISMA-P) were used to conduct the search.^32^ A brief literature search and scoping was conducted in consultation with a librarian and key experts in adolescent trauma to develop and refine the search terms prior to commencing. Final search terms used for the study were mainly: violence, violent crime, abuse (emotional abuse, physical abuse or sexual abuse), school violence, trauma (emotional trauma, post-traumatic stress or trauma reactions), PTSD or post-traumatic stress or post-traumatic stress disorder, anxiety or anxiety disorder, acute stress disorder, depression, conduct disorder, adolescence/adolescent, Africa, African continent, sub-Saharan Africa. The protocol was preregistered on Prospero (CRD42023390724) and no further amendments were required. Systematic literature searches were conducted using MEDLINE (1946–2023), PsycInfo (1806–2023), Web of Science Core Collection (1923–2023) and Global Health (1973–2023) databases between 18 December 2022 and 6 February 2023 and updated on 25 June 2024. A hand citation search was also conducted. Search outcomes relevant to the study were imported to Zotero version 6.0.37 (Roy Rosenzweig Center for History and New Media at George Mason University, Virginia, USA; https://www.zotero.org/download/) and duplicates removed. Corresponding authors of 11 studies were contacted for clarifications (mainly because of unclear context and/or type of violence). Authors who had not responded after 15 days were followed up and their studies were excluded if no response was received after follow-up.

Study selection occurred in three stages: title, abstract, then full text. Titles and abstracts were ‘blindly’ screened by two raters (M.B.L. and S.F.) against the pre-established inclusion and exclusion criteria, and ‘almost perfect’ agreement was achieved (Cohen’s coefficient *k* = 0.87). Twenty-five per cent of the full texts were blindly screened by the two raters and agreement was achieved (Cohen’s *k* = 0.83).^33^ Remaining articles were screened by rater one (M.B.L.). Discrepancies at all stages were discussed and resolved by the two raters. Grey literature was excluded because peer review was the first level of quality assessment. Papers not in the English language were also excluded.

### Data extraction

Rater one (M.B.L.) extracted 100% of data from selected articles (from 5 March 2023) using study characteristics relevant for the study, including (a) study design, (b) sampling demographics and research location, (c) violence and outcome subtypes, (d) mediators/moderators, and (e) association measures.

### Risk of bias (quality) assessment

All studies were quality assessed (by M.B.L. or H.L.) using Kmet et al’s QualSyst tool^34^ and 30% were independently assessed a second time by both raters, but interrater agreement level was poor owing to different interpretations of the rating criteria (Cohen’s *k* = 0.10; *P* = 0.003). Disagreements were discussed and resolved, assessment criteria were clarified and a further 30% were independently assessed a second time, achieving perfect interrater agreement (*k* = 1.00).

### Synthesis

The papers in the current review were heterogeneous, and therefore a narrative synthesis using the framework established by the Economic and Social Research Council (ESRC)^35^ was used to synthesise the findings.

## Results

### Study characteristics

**Fig. 1:**
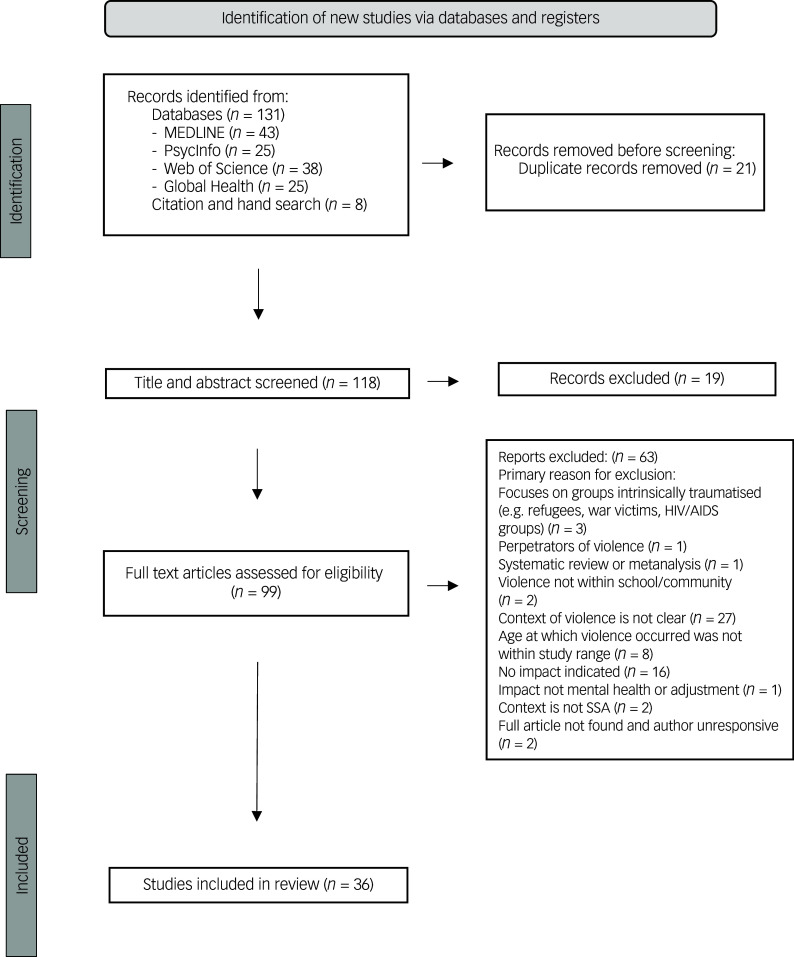
PRISMA flowchart. SSA, sub-Saharan Africa.

This review consisted of 36 studies: two were qualitative and the remainder were quantitative (Fig. 1). The majority were conducted in South Africa (*n* = 23, plus 1 jointly conducted in South Africa and Kenya), followed by Ghana (*n* = 4) and Kenya (*n* = 3). There was evidence suggesting that prevalence rates of community violence varied by region, suggesting that violence exposure in other regions of Africa may begin at lower rates than in South Africa (reported prevalence was 40–45% in Ghana and Malawi^4,36^ versus 60–98% in South Africa^3,28,37,38^). This may provide insight into why more studies found in the current review were conducted in South Africa, which may experience higher rates of violence. In addition, the high concentration of research being on South Africa affected the diversity of the studies in this review. Therefore, it may be beneficial to further investigate how other context-specific factors, such as poverty, inequality, attitudes towards violence, urban versus rural settings, and culture, influence levels of community and school violence, as well as the amount of research conducted on the topic across different regions on the African continent.

### Quality appraisal

The majority of studies (*n* = 24) were methodologically strong based on the QualSyst tool^34^ (score range: 82–95%). The remaining were of adequate (*n* = 1; score: 59%) to good (*n* = 11; score range: 64–77%) quality. No studies were excluded because of poor methodological quality.

### Overview of included studies

The 36 included studies were subjected to a narrative synthesis and results are presented based on themes of areas of functioning affected by exposure to community and/or school violence. Mediating and/or moderating variables were also analysed and presented. In total, 12 studies investigated bullying and violence occurring within the school context. Only 1 of the 12 was qualitative,^39^ and the rest were quantitative.^1,4,26,27,36,38,40–44^ Other types of violence reported in these school based violence studies were sexual, physical and/or emotional violence.^1,27,40^ The remaining 24 studies explored forms of violence exposure that occurred in community contexts, including sexual assault, robbery and other physically aggressive behaviours. Only 1 of these 24 studies was qualitative.^45^ The majority of the 36 studies used non-probability sampling techniques: 7 used purposive,^7,28,39,45–48^ 13 used convenience^3,27,38,40,41,49–56^ and 2 used census sampling.^19,57^ The other 14 studies used probability sampling through random and/or stratified multilevel techniques.^1,4,9,16,24–26,36,37,42,44,58–60^ Broader types of violence in the 24 community studies included violence causing physical harm and/or the threat of physical harm, and sexual violence.

### Themes

#### Impact on psychological functioning

##### Bullying studies

Based on quantitative investigations, bullying victimisation was reported to be linked to depression (including suicidal behaviours), anxiety and post-traumatic stress symptoms (PTSS, i.e. trauma symptoms that do not meet the full diagnostic criteria for a post traumatic stress disorder)/stress at statistically significant levels, with small (depression and post-traumatic stress) and large (anxiety) effect sizes (Supplementary Table B2).^1,36,38,40–44^ Notably, Boyes et al^42^ found small reductions in anxiety and depression symptoms over time for both genders, and some reduction in PTSS for males but not females at statistically significant levels. Penning et al^38^ also found significant associations between bullying and anger among males (who scored lowest of all the Trauma Symptoms Checklist for Children (TSCC-A) subscales). Qualitatively, there were increased internalising symptoms for bullying witnesses, indicating that adolescents may experience cognitive dissonance (Supplementary Table B1). This was a result of understanding that the bullying was wrong but not intervening owing to fear of being the next victim.^39^ Additionally, they experienced feelings of shame and guilt for not defending the victim, for whom they felt compassion and empathy. Adolescents in this study also described PTSS (difficulties concentrating due to intrusive thoughts and flashbacks), which further increased distress as flashbacks were re-traumatising. This exacerbated feelings of sadness, anger, restlessness, shock and worry about being the next victim.^39^ These findings suggest that adolescents who experienced bullying were two to three times more likely to develop internalising symptoms, and, although there may be some reduction in these symptoms over time, some adolescents continued to struggle for prolonged periods.

##### Physical violence/threat of physical harm

For violence occurring within schools, two studies found that only physical abuse by teachers had significant associations with depression,^27,40^ whereas Nkuba et al^1^ noted that, overall, physical violence by teachers did not have an impact at a statistically significant level on mental health outcomes. These findings should be interpreted with caution as Nkuba et al’s study compared the impact of both parental and teacher violence on adolescents’ broader mental health outcomes and found that although physical violence by teachers showed some statistical significance, this statistical significance was absent when violence by parents was controlled for within the model of analysis. Exposure to broader community violence, involving threat to/actual physical harm (witnessing and victimisation) was found to have a significant impact on mental health. There were varying associations between the reported rates for depression, anxiety and post-traumatic stress disorder (PTSD), although rates of comorbidity were high in all studies investigating multiple outcomes. Findings further suggest that those reporting more frequent exposure to violence or those with exposure to multiple forms of violence have increased risk for the above mental health outcomes.^9,16,25,47,56,57^ Oladeji et al^58^ reported lowest odds of an association between violent crime exposure and PTSD; however, the study investigated a total of 11 different violence experiences and scored lower on our quality rating assessment compared with most of the above studies.

Similarly, Visser et al’s study^50^ investigating the impact of exposure to community violence on the expression of personality (that is, focusing primarily on measuring whether there are differences in expression of 16 personality factor characteristics for those exposed to interpersonal violence compared with those who have not been exposed) found significant associations between violence exposure and PTSD. A quantitative study also found that perceived stress about neighbourhood safety contributed to PTSD symptoms at statistically significant levels.^53^ Similarly, qualitative investigations reported findings of adolescents struggling with feelings of shame, anger and becoming violent because of bullying by gang members in their community. Both genders reported heightened awareness and anxiety around gangs within their communities, which reduced their feelings of safety.^45^ Conversely, there were participants who described being desensitised to violence and considered it ‘normal’. Lastly, findings on differences in the impact of direct victimisation and witnessing violence varied. Some studies found weaker associations of victimisation with depression than witnessing violence,^24^ whereas others found that direct victimisation significantly predicted depression, anxiety and PTSD.^48^ Additionally, indirect community victimisation significantly predicted perceived stress,^48^ whereas poly-victimisation predicted depression, anxiety and PTSD at statistically significant levels.^16,57^

There were inconsistencies regarding the contribution of trauma load (having cumulative trauma experiences) to PTSD symptoms, with Hiscox et al^37^ reporting that trauma load was significantly associated with increase in PTSD symptoms, whereas Nothling et al^48^ found that trauma load did not predict PTSD symptoms. The difference in findings between the two studies may be because Nothling et al’s sample^48^ were adolescents identified as having trauma experiences, including domestic and sexual violence, whereas Hiscox et al^37^ had a larger random sample that included those with and without violence experiences. It is evident that exposure to violence has an impact on depression, anxiety and, particularly, PTSD. One-third to half of the participants in these studies developed mental disorders following violence exposure. The impact significantly increased with cumulative trauma, in turn increasing suicide risk. There may also be increased risk of desensitisation, which may result in negative life outcomes.

##### Sexual violence

Childhood sexual assault has also been linked to later internalising and externalising symptoms and to re-enactments. Trauma re-enactment involves the tendency to recreate or re-enact past traumas.^61^ Re-enactments include perpetration, self-injury, revictimisation and multiple ‘poly re-enactments’ (two or more of the above re-enactments). In terms of internalising symptoms, childhood sexual assault occurring in school and community contexts was significantly associated with depression, anxiety and PTSD.^7,26,28,49,54,62,63^

#### Impact on behavioural functioning

##### Bullying

Elevated externalising symptoms linked to bullying victimisation included bullying initiation and/or perpetration, conduct problems, tobacco use and violence were found to be at statistically significant levels for both males and females, with moderate to large effect sizes.^4,26,40,42,44,46^ Liang et al^44^ additionally noted between-group differences across various forms of bullying, such as bullies being twice as likely to engage in alcohol use and violent, antisocial and risk-taking behaviour, whereas bully-victims showed 3–5 times higher odds of vandalism and suicidal behaviour than either bully or victim groups. These findings suggest that adolescents who experience bullying victimisation may be significantly more likely to present with bullying perpetration, conduct problems and substance use.

##### Physical violence

Physical violence perpetrated by teachers was associated with becoming a bully in males only,^40^ and physical violence experienced in the broader community was significantly associated with behavioural problems such as aggression, conduct problems and substance use in both genders (worse in males).^26,49,51^ Additionally, Stansfeld et al^16^ noted that adolescents who used drugs and alcohol were twice as likely to attempt suicide, whereas Cluver et al^25^ found no mediating relationship between drug or alcohol misuse and suicidal behaviours. Cluver et al^25^ investigated specifically suicidal behaviour, reporting depression, anxiety and PTSD as mediators and not outcomes. This may have resulted in their finding that drug/alcohol use did not mediate suicidal behaviour. These findings indicate that threat of or actual physical violence may increase adolescents’ risk of self-harm and violence perpetration against others.

##### Sexual violence

Externalising symptoms such as aggression and conduct problems were found to have significant positive correlations with childhood sexual assault.^7,26,63^

#### Impact on social functioning

##### Bullying

According to Ameli et al,^40^ females who were victims of bullying were almost twice as likely to develop attitudes that condone violence against and rape of women, although males did not present with similar outcomes. There was also evidence that bullying victimisation results in increased experiences of loneliness among adolescents.^36^ This suggests bullying has both immediate and longer-term risks including risk to further violence experiences.

##### Physical violence

Some adolescents had increased risk of negative life events. For example, South African adolescents experienced break-ups of romantic relationships with a partner but Kenyans did not.^49^ Notably, both groups had similar violence exposure but South Africans were more affected, albeit the study’s authors were unable to explain differences in impact. Adolescents who lived in high-violence environments also experienced stigmatised identities, which included being associated with prostitution as well as drug and alcohol use.^45^ This led to adolescents being bullied (mostly verbally). These results highlight the increased risks experienced by adolescents in these contexts as it extends beyond physical and mental well-being and can further result in peer exclusion and/or victimisation.

##### Sexual violence

Penning & Collings^19^ found that community childhood sexual assault was significantly associated with revictimisation and poly re-enactments, with re-enactments being up to twice as likely than in those with no history of sexual assault.

#### Impact on academic performance

##### Bullying

According to Adewoye & du Plessis,^39^ adolescents who experience bullying struggled with PTSS, such as difficulties concentrating due to intrusive thoughts and flashbacks that affected them in school. Additionally, the uncertainty of their own likelihood of being bullied affected their interest in school and attendance.

##### Physical violence/physical threat and sexual violence

Seedat et al^49^ reported that adolescents exposed to violence within their communities performed lower than expected on school tests and examinations. Furthermore, Brown^54^ highlights that the cognitive symptoms related to PTSD found among adolescents exposed to verbal, physical and sexual violence within their communities resulted in low concentration and interest in school work. Evidently, violence exposure not only affects mental, behavioural, emotional and psychological functioning, as these consequences further affect students’ academic performance and increase reluctance to engage at school.

### Mediators or moderators of effects of violence exposure on mental health and adjustment outcomes

#### Bullying

Several variables were found to partially mediate and moderate the impact of bullying on adolescent mental health and life outcomes. Kim et al^4^ found significant differences in peer affiliation, bullying victimisation and loneliness between the group reporting tobacco and alcohol use compared with the group that did not. Adolescents who had fewer peer affiliations were more likely to be bullied. Those who were bullied and had increased levels of loneliness were significantly more likely to use tobacco and alcohol, compared with those who did not. There was also a direct association between peer affiliation and tobacco use, with those who engaged in tobacco use having fewer peer affiliations compared with those who did not.^4^ Similarly, Diallo et al^43^ found that adolescents who had close friends had lower odds of suicide attempts, further highlighting moderating effects of peer affiliations.

#### Physical violence/physical threat

There were four studies investigating factors that moderate the effect of exposure to physical violence on mental health and adjustment problems. O’Donnell et al,^56^ Stansfeld et al^16^ and Wado et al^60^ investigated interpersonal (parents’ warmth and/or social support) and environmental (school climate) moderators, and Fincham et al^53^ explored intrapersonal factors (resilience). It was found that a positive school climate significantly moderated the effects of mild to moderate exposure to violence on PTSS levels for both witnessing and direct victimisation; however, these moderating effects were not evident for higher levels of exposure.^56^ The findings for moderating effects of social support on exposure to violence varied. O’Donnell et al^56^ found parental warmth to have no significant moderating effects. Conversely, Wado et al^60^ found a significant moderating effect of parental connectedness on self-reported depression, whereas Stansfeld et al^16^ noted a minimal moderating effect for social support, albeit no statistical significance was reported. Although O’Donnell et al^56^ and Stansfeld et al^16^ had a stronger quality rating, Wado et al^60^ was the most recent study, had more participants (followed by Stansfeld et al^16^) and was conducted across two countries (South Africa and Kenya), which may explain the discrepancies between the studies. Lastly, Fincham et al^53^ found no association between resilience, perceived stress or exposure to community violence, and PTSD symptoms. Of note, this study investigated various adverse childhood experiences, and resilience was found to have a statistically significant moderating effect for childhood abuse and neglect on the development of PTSD symptoms. Fincham et al noted that resilience may lose its moderating effect when adolescents are in environments in which they experience acute stress and trauma, which do not allow for processing of negative information. Although these findings were inconsistent, they suggest that there may be factors that moderate the effects of being exposed to violence.

#### Sexual violence

Several moderator variables and one mediating variable were found between childhood sexual assault and mental health. Syengo-Mutisya et al^63^ reported that the family’s ability to sort out disagreements between parent and child moderated psychiatric morbidity following childhood sexual assault. Notably, the severity of depressive symptoms was found to significantly reduce over time (measured at 4 months and 1 year) from mild to moderate at baseline to minimal following intervention (the nature of the intervention is not stated, but the study was from the Gender Based Violence Recovery Centre in Nairobi).^7^ Children below 16 years continued to experience depression at statistically significant levels despite comprehensive and specialised care, although there was no indication why this should be.^7^ On the other hand, negative appraisals following childhood sexual assault were found to predict re-enactments at statistically significant levels.^19^ These studies highlight the moderating effects of social support and early interventions and the mediating effects of trauma appraisal.

Overall, there is both qualitative and quantitative evidence that community and school violence exposure has a significant impact on adolescent mental health and adjustment outcomes, resulting in internalising and externalising symptoms as well as decreased school attendance and impaired social functioning. Furthermore, exposure to community and school violence creates a breeding ground for further violence perpetration, re-victimisation and/or attitudes that condone violence, creating an ongoing cycle of violence perpetration and exposure. Although slight differences may be found across different exposure types (i.e. across violence categories as well as being a victim or witness), adolescents can be said to experience community and school violence consistently and continually, and the impact of this violence is often prolonged. Lastly, bullying victimisation and related mental health and adjustment outcomes were found to be influenced by interpersonal, intrapersonal and environmental factors that mediate and/or moderate exposure to community and school violence, albeit these variables can be limited by high levels of exposure.

## Discussion

The current review produced findings that were consistent with previous reviews (which focused largely on the Global North) that investigated adolescent exposure to various forms of violence. Reviews that explored bullying victimisation, sexual violence and physical violence occurring in schools and communities consistently found high levels of internalising and externalising symptoms, with females reporting higher levels of internalising symptoms compared with males, who were more likely to present with externalising behaviours,^64,65^ which is similar to our findings. There was also evidence that proximity to violence was an important determinant of the outcomes of the exposure. Adolescents who were directly victimised were at greater risk for more severe symptoms than those who witnessed violence, albeit witnessing was still associated with adverse outcomes. Similarly, Miliauskas et al^13^ observed the same outcomes in their review, highlighting that the longitudinal studies provide stronger causal evidence that confirms the association between violence exposure and mental disorders. Notably, the current review also found evidence suggesting that factors linked to social integration (such as developing attitudes that condone violence and stigmatise identities) are crucial in influencing the levels of violence exposure and the resulting impact that was not evident in other reviews. This draws attention to the need to understand more context-specific factors that may exacerbate or ameliorate the impact of violence exposure in various contexts to inform interventions.

Studies in the current review highlighted that males experienced higher levels of community violence than females (who were more likely to experience sexual assault). Males presented with more externalising and females presented with more internalising symptoms.^3,9,16,26,48,49,53^ Additionally, ethnic and age/grade cohort differences were also found in the impact of exposure to community violence, with females, older adolescents, those in higher grades, as well as Black and adolescents from a Mixed ethnic background, being more vulnerable to being significantly impacted by exposure to violence.^16,24,53^ These findings were consistent with other reviews^13^ drawing attention to the increased vulnerability some adolescent groups may have over others.

The most significant mediators and moderators for the effects of violence were intrapersonal, social and environmental factors. Adolescents who had more peer, sibling and parental support had more positive outcomes compared with those who did not.^16,56,60^ Other factors included school climate and resilience, which lost its mediating effect in the face of high levels of violence exposure.^53^ The importance of moderating factors such as family support was also highlighted in other reviews as being crucial for adolescents who are exposed to violence.^13,65^ Lastly, individual factors such as resilience, reframing as well as desensitisation played a moderating role in studies in the current review. However, caution should be taken when considering coping strategies such as desensitisation which, although reducing psychological symptoms such as depression, anxiety and PTSD, can further contribute to increased levels of externalising symptoms such as violence perpetration.^13^ Further investigation and understanding of these mediator and moderator variables, as well as their limitations across specific groups, may provide insight into the development of interventions aimed at ameliorating the impact of exposure to community and school violence.

### Strengths and limitations

There are a number of strengths evident in the current review. The review broadly investigated the impact of community violence on mental health and adjustment of adolescents. The search terms used allowed the capture of a wide range of possible articles. Although this resulted in a significant number of articles to screen, it may have ensured that possible articles related to this topic were not missed. This review also contributes to a limited body of knowledge about the topic in LMICs such as countries on the African continent. There are also several limitations. The articles included were restricted to those published in English, which may have excluded some relevant papers. However, there were very few non-English-language studies on the topic. The grey literature was not searched as we relied on the peer review process as the first level of quality assessment. Lastly, although the methodological quality of most of the studies was high, some studies failed to meet several criteria on the methodological quality assessment tool used.

### Future research

The current review aimed to investigate the development (and progression) of mental health and adjustment outcomes of adolescents exposed to violence in their schools and communities. Future research investigating these factors would benefit from case–control studies with strong controls for confounding variables and longitudinal studies. Most of the studies in the review investigating the above factors were cross-sectional, so causation could not be inferred. Only three longitudinal studies were found in the current review and these had methodological issues, including poor reliability of measures of both violence exposure and the resulting internalising and externalising symptoms. These methodological issues were reported to be problems related to social desirability found in self-report measures, which may have been amplified through the use of interviews as the primary method of data collection, as in the case of Boyes et al^42^ and Cluver et al.^25^ This suggests that the combined use of interviews and questionnaires may be beneficial. Although these studies had a high retention rate, some of the participants that could not be traced were noted to be among the most vulnerable to poor mental health and adjustment outcomes, which may have resulted in an underestimation of the strength of the observed relationship.^25,42^ In addition, although some studies in our review indicated mediating and moderating factors, they did not investigate modifiable psychological processes that mediate the development of mental health and adjustment problems, which is crucial for informing interventions. Future research may benefit from the use of both qualitative and quantitative methods to aid the development and evaluation of interventions which deploy early identification and secondary prevention interventions, which could mitigate effects of exposure to violence for youth in high risk contexts and emerging economies which face additional economic challenges. It is also recommended that future research investigate the current landscape of interventions and best practices within the topic. This can include synthesising knowledge on the reach and impact of social media and/or the mediating impact of social media following violence exposure. Lastly, it would also be beneficial to build insights into agencies (including local and international government, as well as non-government) involved in prevention and/or amelioration of the impact of violence exposure within this population to further build on strengthening evidence practices and reach.

### Clinical implications

Several clinical implications can be drawn from the findings of the current review. First, given the high rates of continued violence exposure in communities and schools in African countries, especially South Africa, there is a need for primary prevention of violence exposure. In schools where teachers still engage in corporal punishment, this can involve educating teachers on alternative methods of discipline and the consequences of physical abuse.^27^ Second, in contexts where adolescents are already exposed to violence, there is a critical need for identification of at-risk individuals and early secondary prevention interventions before the resulting challenges develop and progress into young adulthood. These secondary prevention interventions can include regular school screening for mental health challenges among adolescents, and the development of interventions involving the entire school that target modifiable mediating and/or moderating factors.^41,42^ Essential to the effective and cost-effective deployment of such measures are studies investigating predictors, moderators and mediators of the effects of violence on later mental health. Building on this review, studies on support and interventions for individuals who were exposed to violence in their adolescence and developed challenges that continue into their adulthood are likely to be crucial to prevent further progression of challenges and to improve quality of life.^17,18^ Lastly, it is paramount that the interventions suggested above are developed based on evidence-based approaches like cognitive–behavioural therapy (CBT) that are culturally responsive and sensitive to the contexts they are developed for.

## Supporting information

Lupindo et al. supplementary material Lupindo et al. supplementary material

## Data Availability

The data that support the findings of this study, including the search strategy, various levels of screening, data extraction and details of all articles used for this review, are available on request from the corresponding author.

## Supplementary material

The supplementary material is available online at https://doi.org/10.1192/bji.2025.10043.
