# Supplementary material for: Impact of exposure to community and school violence during adolescence in the African context: systematic review
Source: BJPsych Int. 2025 Sep 1;22(4):120–30. doi: 10.1192/bji.2025.10043 (PMC13054169; doi:10.1192/bji.2025.10043)
Supplement: Lupindo et al. supplementary material [file S2056474025100433sup001.docx]

**Appendix A. PRISMA-P Flow Chart and Search Strategy**

**FIGURE A1:** PRISMA flowchart (Shamseer et al., 2015)

**Identification of new studies via databases and registers**

Records identified from*:

**Databases** (n = 131)

- Medline (n = 43)
- PsycInfo (n = 25)
- Web of Science (n = 38)
- Global Health (n = 25)

**Citation and hand search** (n = 8)

Records removed before screening:

Duplicate records removed (n = 21)

Identification

Records excluded (n = 19)

Title and abstract screened (n = 118)

Reports excluded: (n = 63)

**Primary reason for exclusion**:

Focuses on groups intrinsically traumatized (e.g. refugees, war victims, HIV/AIDS groups) (n = 3)

Perpetrators of violence (n = 1)

Systematic review or metanalysis (n = 1)

Violence not within school/ community

(n = 2)

Context of violence is not clear (n = 27)

Age at which violence occurred was not within study range (n = 8)

No impact indicated (n = 16)

Impact not mental health or adjustment (n =1)

Context is not SSA (n = 2)

Full article not found and author unresponsive (n = 2)

Screening

Full text articles assessed for eligibility

(n = 99)

Included

Studies included in review (n = 36)

*Note. Abbreviations: n = Number*

**Table A1: PsycInfo Search Strategy**

| **Database** | **Search Strategy** |
| --- | --- |
| PscyInfo | ((“Gun Violence” or “Violence” or “School violence” or “Sexual Violence” or “violent crime”) not (“video games” or “computer games)] or (Emotional abuse” or “Physical abuse” or “verbal abuse” or “sexual abuse”))  and  ((“stress and trauma related disorders” or “trauma reactions” or “emotional trauma” or trauma) or (PTSD or “post traumatic stress” or “trauma reactions” or anxiety or "acute stress disorder” or depression or “conduct disorder”))  and  adol*  and  ((“Africa South of the Sahara" or "Sub Saharan Africa" or Angola or Benin or Botswana or "Burkina Faso" or Burundi or Cameroon or "Cape Verde" or "Central African Republic" or Chad or Comoros or Congo or Brazzaville or "Cote dIvoire" or Djibouti or "Equatorial Guinea" or Eritrea or Ethiopia or Gabon or Gambia or Ghana or Guinea or Bissau or Kenya or Lesotho or Liberia or Madagascar or Malawi or Mali or Mauritania or Mauritius or Mozambique or Namibia or Niger or Nigeria or Rwanda or "Sao Tome e Principe" or Senegal or Seychelles or "Sierra Leone" or Somalia or "South Africa" or "South Sudan" or Sudan or Swaziland or Tanzania or Togo or Uganda or "Western Sahara" or Zaire or Zambia or Zimbabwe) OR ("western cape" or "eastern cape" or "kwazulu natal" or " gauteng" or "mpumalanga" or "free state" or "north west" or limpopo or "northern cape”) or "western cape" or "eastern cape" or "kwazulu natal" or " gauteng" or "mpumalanga" or "free state" or "north west" or limpopo or "northern cape")) |
| Medline | ((“Gun Violence” or “Violence” or “School violence” or “Sexual Violence” or violence) not (“video games” or “computer games)] or (Emotional abuse” or “Physical abuse” or “verbal abuse” or “sexual abuse”))  and  ((“stress and trauma related disorders” or “trauma reactions” or “emotional trauma” or trauma) or (PTSD or “post traumatic stress” or “post traumatic stress disorder” or depression or “conduct disorder”))  and  adol*  and  ((“Africa South of the Sahara" or "Sub Saharan Africa" or Angola or Benin or Botswana or "Burkina Faso" or Burundi or Cameroon or "Cape Verde" or "Central African Republic" or Chad or Comoros or Congo or Brazzaville or "Cote dIvoire" or Djibouti or "Equatorial Guinea" or Eritrea or Ethiopia or Gabon or Gambia or Ghana or Guinea or Bissau or Kenya or Lesotho or Liberia or Madagascar or Malawi or Mali or Mauritania or Mauritius or Mozambique or Namibia or Niger or Nigeria or Rwanda or "Sao Tome e Principe" or Senegal or Seychelles or "Sierra Leone" or Somalia or "South Africa" or "South Sudan" or Sudan or Swaziland or Tanzania or Togo or Uganda or "Western Sahara" or Zaire or Zambia or Zimbabwe) OR ("western cape" or "eastern cape" or "kwazulu natal" or " gauteng" or "mpumalanga" or "free state" or "north west" or limpopo or "northern cape”) or ("western cape" or "eastern cape" or "kwazulu natal" or " gauteng" or "mpumalanga" or "free state" or "north west" or limpopo or "northern cape")) |
| Global Health | ((“Gun Violence” or “Violence” or “School violence” or “Sexual Violence” or “violent crime”) not (“video games” or “computer games)] or (Emotional abuse” or “Physical abuse” or “verbal abuse” or “sexual abuse”))  and  ((Trauma or “stress and trauma related disorders” or “trauma reactions” or “emotional trauma”) or (PTSD or “post traumatic stress” or anxiety or "anxiety disorder” or depression or “conduct disorder”))  and  adol*  and  ((“Africa South of the Sahara" or "Sub Saharan Africa" or Angola or Benin or Botswana or "Burkina Faso" or Burundi or Cameroon or "Cape Verde" or "Central African Republic" or Chad or Comoros or Congo or Brazzaville or "Cote dIvoire" or Djibouti or "Equatorial Guinea" or Eritrea or Ethiopia or Gabon or Gambia or Ghana or Guinea or Bissau or Kenya or Lesotho or Liberia or Madagascar or Malawi or Mali or Mauritania or Mauritius or Mozambique or Namibia or Niger or Nigeria or Rwanda or "Sao Tome e Principe" or Senegal or Seychelles or "Sierra Leone" or Somalia or "South Africa" or "South Sudan" or Sudan or Swaziland or Tanzania or Togo or Uganda or "Western Sahara" or Zaire or Zambia or Zimbabwe) OR ("western cape" or "eastern cape" or "kwazulu natal" or " gauteng" or "mpumalanga" or "free state" or "north west" or limpopo or "northern cape” or "western cape" or "eastern cape" or "kwazulu natal" or " gauteng" or "mpumalanga" or "free state" or "north west" or limpopo or "northern cape")) |
| Web of Science (Core Collection) | ((“Gun Violence” or “Violence” or “School violence” or “Sexual Violence” or “violent crime”) or (Emotional abuse” or “Physical abuse” or “sexual abuse”)) AND ((PTSD or “post traumatic stress” or “post traumatic stress disorder) or anxiety or "anxiety disorder” or “acute stress disorder” or depression or “conduct disorder”) or (Trauma or “stress and trauma related disorders” or “trauma reactions” or “emotional trauma”))  AND ((“Africa South of the Sahara" or "Sub Saharan Africa" or Angola or Benin or Botswana or "Burkina Faso" or Burundi or Cameroon or "Cape Verde" or "Central African Republic" or Chad or Comoros or Congo or Brazzaville or "Cote dIvoire" or Djibouti or "Equatorial Guinea" or Eritrea or Ethiopia or Gabon or Gambia or Ghana or Guinea or Bissau or Kenya or Lesotho or Liberia or Madagascar or Malawi or Mali or Mauritania or Mauritius or Mozambique or Namibia or Niger or Nigeria or Rwanda or "Sao Tome e Principe" or Senegal or Seychelles or "Sierra Leone" or Somalia or "South Africa" or "South Sudan" or Sudan or Swaziland or Tanzania or Togo or Uganda or "Western Sahara" or Zaire or Zambia or Zimbabwe) OR ("western cape" or "eastern cape" or "kwazulu natal" or " gauteng" or "mpumalanga" or "free state" or "north west" or limpopo or "northern cape”) or ("western cape" or "eastern cape" or "kwazulu natal" or " gauteng" or "mpumalanga" or "free state" or "north west" or limpopo or "northern cape")) |

**Appendix B. Inclusion and Exclusion Criteria**

**
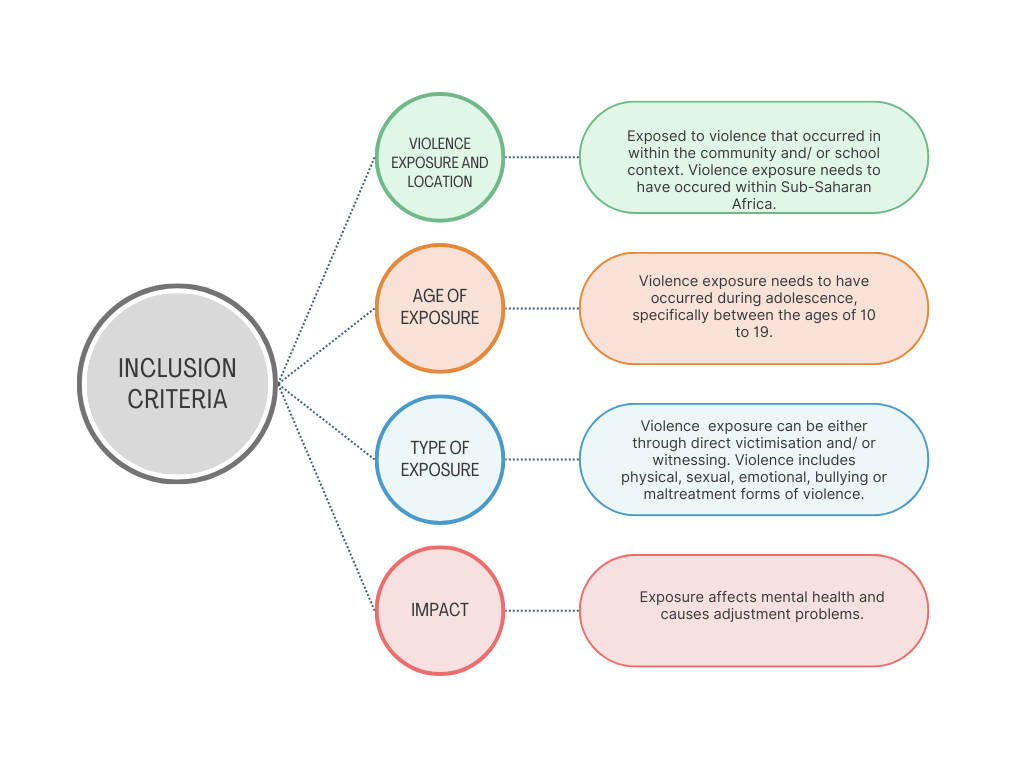
**

**Figure B1: Inclusion Criteria**

**
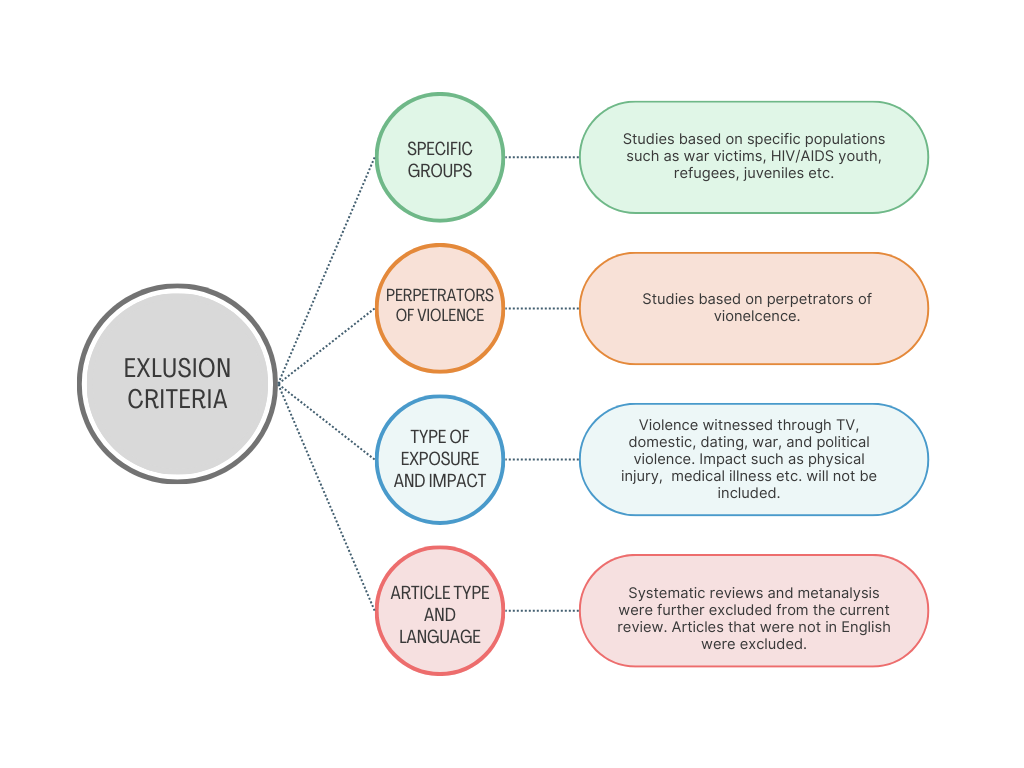
**

**Figure B2: Exclusion Criteria**

**Appendix C. Inter rater clarifications for QualSyst tool**

**Table C1: Inter rater clarifications for QualSyst tool**

|  | **Criterion 1** | **Criterion 2** | **Criterion 3** | **Criterion 4** | **Criterion 5** | **Criterion 6** | **Criterion 7** | **Criterion 8** | **Criterion 9** | **Criterion 10** | **Criterion 11** | **Criterion 12** | **Criterion 13** | **Criterion 14** |
| --- | --- | --- | --- | --- | --- | --- | --- | --- | --- | --- | --- | --- | --- | --- |
| **Kmet tool:** | **Question / objective sufficiently described?** | **Study design evident and appropriate?** | **Method of subject/comparison group selection *or* source of information/input variables described and appropriate?** | **Subject (and comparison group, if applicable) characteristics sufficiently described?** | **If interventional and random allocation was possible, was it described?** | **If interventional and blinding of investigators was possible, was it reported?** | **If interventional and blinding of subjects was possible, was it reported?** | **Outcome and (if applicable) exposure measure(s) well defined and robust to measurement / misclassification bias? Means of assessment reported?** | **Sample size appropriate?** | **Analytic methods described/ justified and appropriate?** | **Some estimate of variance is reported for the main results?** | **Controlled for confounding?** | **Results reported in sufficient detail?** | **Conclusions supported by the results?** |
|  |  | **For 2 points they need to explicitly name the design. 1 point if design is evident but not explictly stated.** | **If only state "random sampling" but don't describe how (e.g. don't say what criteria or what tool used) = only 1 points. If state from x location, but don't say HOW got the sample (e.g. if don't specifiy whether it was random, self select etc) = still only 1 point.** | **Inclusion/exclusion criteria comes under criterion 3, not under 4. This critiera is only for subject characteristics described.** | **N/A excluded** | **N/A excluded** | **N/A excluded** |  | **If they did not say how they came up with sample size / have no power analysis in general = 1 point.** | **If they don't explicitly in the methods section what analytical methods they use = 1 point** |  | **Not N/A. Studies should control for confounding, if they don't mention it or don't control = 0** |  | **If they make clear assumptions in the discussion/conclusion that correlation infers causation, then = 1 point. If they note in the limitations/discussion that correlations cannot conclude causation then = 2 points (even if the abstrac or other parts hint at causation). If broad conclusion are drawn ONLY about whole country/city without mentioning that their sample was more specific = 1 point. If they note conclusions based on their specific population (e.g. city) AND bigger picture (e.g. country) = can give 2 points.** |

**Appendix D. QualSyst Ratings for Included Studies**

**Table D1: Qualitative Studies**

| **Author, Year** |  | **Criterion 1** | **Criterion 2** | **Criterion 3** | **Criterion 4** | **Criterion 5** | **Criterion 6** | **Criterion 7** | **Criterion 8** | **Criterion 9** | **Criterion 10** | **Total Sum** | **Total possible sum (20 total possible)** | **Summary score** |
| --- | --- | --- | --- | --- | --- | --- | --- | --- | --- | --- | --- | --- | --- | --- |
|  | **Kmet tool:** | **Question / objective sufficiently described?** | **Study design evident and appropriate?** | ***Context for the study clear?*** | **Connection to a theoretical framework / wider body of knowledge?** | **Sampling strategy described, relevant and justifi ed?** | **Data collection methods clearly described and systematic?** | **Data analysis clearly described and systematic?** | **Use of verifi cation procedure(s) to establish credibility?** | **Conclusions supported by the results?** | ***Reflexivity of the account?*** |  |  | Sum of points awarded to the study divided by the number possible to achieve (i.e. without non-applicable items) |
|  |  | **Rating: 2=yes, 1=partial, 0=no, N/A** | **Rating: 2=yes, 1=partial, 0=no, N/A** | **Rating: 2=yes, 1=partial, 0=no, N/A** | **Rating: 2=yes, 1=partial, 0=no, N/A** | **Rating: 2=yes, 1=partial, 0=no, N/A** | **Rating: 2=yes, 1=partial, 0=no, N/A** | **Rating: 2=yes, 1=partial, 0=no, N/A** | **Rating: 2=yes, 1=partial, 0=no, N/A** | **Rating: 2=yes, 1=partial, 0=no, N/A** | **Rating: 2=yes, 1=partial, 0=no, N/A** |  |  |  |
| Adewoye and du Plessis (2021) |  | 2 | 2 | 2 | 2 | 1 | 2 | 2 | 2 | 2 | 0 | 17 | 20 | 0,85 |
| Scorgie et al. (2018) |  | 2 | 2 | 2 | 1 | 2 | 2 | 2 | 0 | 2 | 0 | 15 | 20 | 0,75 |
|  |  | **4** | **4** | **4** | **3** | **3** | **4** | **4** | **2** | **4** | **0** | 32 |  |  |

**Table D2: Quantitative Studies**

| **Author, Year** |  | **Criterion 1** | **Criterion 2** | **Criterion 3** | **Criterion 4** | **Criterion 5** | **Criterion 6** | **Criterion 7** | **Criterion 8** | **Criterion 9** | **Criterion 10** | **Criterion 11** | **Criterion 12** | **Criterion 13** | **Criterion 14** | **Total sum** | **Total possible sum (22 total possible)** | **Summary score** | **Final Agreed Score** |
| --- | --- | --- | --- | --- | --- | --- | --- | --- | --- | --- | --- | --- | --- | --- | --- | --- | --- | --- | --- |
|  | **Kmet tool:** | **Question / objective sufficiently described?** | **Study design evident and appropriate?** | **Method of subject/comparison group selection *or* source of information/input variables described and appropriate?** | **Subject (and comparison group, if applicable) characteristics sufficiently described?** | **If interventional and random allocation was possible, was it described?** | **If interventional and blinding of investigators was possible, was it reported?** | **If interventional and blinding of subjects was possible, was it reported?** | **Outcome and (if applicable) exposure measure(s) well defined and robust to measurement / misclassification bias? Means of assessment reported?** | **Sample size appropriate?** | **Analytic methods described/ justified and appropriate?** | **Some estimate of variance is reported for the main results?** | **Controlled for confounding?** | **Results reported in sufficient detail?** | **Conclusions supported by the results?** |  |  | Sum of points awarded to the study divided by the number possible to achieve (i.e. without non-applicable items) |  |
|  |  | **Rating: 2=yes, 1=partial, 0=no, N/A** | **Rating: 2=yes, 1=partial, 0=no, N/A** | **Rating: 2=yes, 1=partial, 0=no, N/A** | **Rating: 2=yes, 1=partial, 0=no, N/A** | **Rating: 2=yes, 1=partial, 0=no, N/A** | **Rating: 2=yes, 1=partial, 0=no, N/A** | **Rating: 2=yes, 1=partial, 0=no, N/A** | **Rating: 2=yes, 1=partial, 0=no, N/A** | **Rating: 2=yes, 1=partial, 0=no, N/A** | **Rating: 2=yes, 1=partial, 0=no, N/A** | **Rating: 2=yes, 1=partial, 0=no, N/A** | **Rating: 2=yes, 1=partial, 0=no, N/A** | **Rating: 2=yes, 1=partial, 0=no, N/A** | **Rating: 2=yes, 1=partial, 0=no, N/A** |  |  |  |  |
| Ameli et al. (2017) |  | 2 | 2 | 2 | 2 | N/A | N/A | N/A | 2 | 1 | 2 | 2 | 0 | 2 | 2 | 19 | 22 | 0,86 | Resolved |
| Arhin et al. (2019) |  | 2 | 2 | 1 | 2 | N/A | N/A | N/A | 2 | 1 | 2 | 2 | 0 | 2 | 2 | 18 | 22 | 0,82 |  |
| Bach and Louw (2010) |  | 2 | 1 | 1 | 2 | N/A | N/A | N/A | 2 | 1 | 1 | 2 | 0 | 2 | 2 | 16 | 22 | 0,73 |  |
| Boyes et al. (2014) |  | 2 | 2 | 2 | 2 | N/A | N/A | N/A | 2 | 1 | 2 | 2 | 0 | 2 | 2 | 19 | 22 | 0,86 |  |
| Brown (2009) |  | 2 | 2 | 2 | 2 | N/A | N/A | N/A | 2 | 1 | 2 | 2 | 0 | 2 | 1 | 18 | 22 | 0,82 |  |
| Collings et al. (2014) |  | 2 | 2 | 1 No sampling technique or inclusion and exclusion criteria | 2 | N/A | N/A | N/A | 2 | 1 | 1 no mention of analytical methods and appropriateness | 0 | 2 | 2 | 2 | 17 | 22 | 0,77 | Resolved |
| Cluver et al. (2015) |  | 2 | 2 | 2 | 2 | N/A | N/A | N/A | 2 | 1 | 2 | 2 | 2 | 2 | 2 | 21 | 22 | 0,95 |  |
| Diallo et al. (2023) |  | 1 | 2 | 2 | 2 | N/A | N/A | N/A | 2 | 1 | 2 | 0 | 2 | 2 | 1 | 17 | 22 | 0,77 |  |
| Du Plessis et al. (2015) |  | 2 | 1 no study design indicated | 2 | 2 | N/A | N/A | N/A | 2 | 1 no power analysis (same for all of them) | 2 | 2 | 2 | 2 | 2 | 20 | 22 | 0,91 | Resolved |
| Ensink et al. (1997) |  | 2 | 2 | 1 | 1 | N/A | N/A | N/A | 2 | 1 | 2 | 0 | 0 | 2 | 2 | 15 | 22 | 0,68 |  |
| Esterhuyse et al. (2007) |  | 2 | 1 | 1 | 2 | N/A | N/A | N/A | 2 | 1 | 2 | 2 | 0 | 2 | 2 | 17 | 22 | 0,77 |  |
| Fakunmoju and Bammeke (2015) |  | 2 | 2 | 1 | 2 | N/A | N/A | N/A | 2 | 1 | 2 | 2 | 2 | 2 | 2 | 20 | 22 | 0,91 |  |
| Fincham, Altes, Stein and Seedat (2009) |  | 2 | 2 | 1 | 2 | N/A | N/A | N/A | 2 | 1 | 2 | 2 | 2 | 2 | 2 | 20 | 22 | 0,91 |  |
| Hiscox et al. (2021) |  | 2 | 2 | 1 | 2 | N/A | N/A | N/A | 2 | 1 | 2 | 2 | 2 | 2 | 2 | 20 | 22 | 0,91 |  |
| Kaminer, et al. (2013) |  | 2 | 1 | 1 | 2 | N/A | N/A | N/A | 2 | 1 | 2 | 2 | 2 | 2 | 2 | 19 | 22 | 0,86 |  |
| Kim et al. (2018) |  | 2 | 2 | 2 | 2 | N/A | N/A | N/A | 2 | 1 | 2 | 2 | 2 | 2 | 2 | 21 | 22 | 0,95 |  |
| Liang et al. (2007) |  | 2 | 2 | 2 | 2 | N/A | N/A | N/A | 2 | 1 | 2 | 0 | 2 | 2 | 2 | 19 | 22 | 0,86 |  |
| Martin, Revington and Seedat (2013) |  | 2 | 2 | 2 | 2 | N/A | N/A | N/A | 2 | 1 | 2 | 2 | 0 | 2 | 2 | 19 | 22 | 0,86 |  |
| Mutavi at al. (2018) |  | 2 | 2 | 2 | 2 | N/A | N/A | N/A | 2 | 1 | 2 | 0 | 0 | 2 | 2 | 17 | 22 | 0,77 |  |
| Nkuba et al. (2018) |  | 2 | 1 no study design indicated | 2 | 2 | N/A | N/A | N/A | 2 | 1 | 2 | 2 | 2 | 2 | 1 claims to have a nationally representative sample although sample size is small (700) | 19 | 22 | 0,86 | Resolved |
| Nothing et al. (2017) |  | 2 | 2 | 2 | 2 | N/A | N/A | N/A | 2 | 1 | 2 | 2 | 2 | 2 | 2 | 21 | 22 | 0,95 |  |
| Nyarko et al. (2020) |  | 2 | 2 | 1 | 2 | N/A | N/A | N/A | 2 | 1 | 2 | 2 | 2 | 2 | 2 | 20 | 22 | 0,91 |  |
| O’Donnel et al. (2011) |  | 2 | 1 | 2 | 2 | N/A | N/A | N/A | 2 | 1 | 2 | 2 | 2 | 2 | 2 | 20 | 22 | 0,91 |  |
| Owusu et al. (2011) |  | 2 | 1 | 2 | 2 | N/A | N/A | N/A | 2 | 1 | 2 | 2 | 2 | 2 | 2 | 20 | 22 | 0,91 |  |
| Peltzer (1999) |  | 2 | 1 | 1 | 2 | N/A | N/A | N/A | 2 | 1 | 1 | 0 | 0 | 2 | 2 | 14 | 22 | 0,64 |  |
| Penning et al. (2010) |  | 2 | 2 | 2 | 2 | N/A | N/A | N/A | 2 | 1 | 2 | 2 | 2 | 2 | 2 | 21 | 22 | 0,95 |  |
| Penning and Collings (2014) |  | 2 | 1 | 1 | 1 | N/A | N/A | N/A | 2 | 1 | 2 | 0 | 2 | 2 | 2 | 16 | 22 | 0,73 |  |
| Schwartz et al. (2021) |  | 2 | 1 | 1 | 2 | N/A | N/A | N/A | 2 | 1 | 2 | 2 | 2 | 2 | 2 | 19 | 22 | 0,86 |  |
| Seedat et al. (2004) |  | 2 | 1 | 1 | 2 | N/A | N/A | N/A | 2 | 1 | 2 | 0 | 0 | 2 | 2 | 15 | 22 | 0,68 |  |
| Seyengo et al. (2008) |  | 2 | 2 | 2 | 2 | N/A | N/A | N/A | 1 | 1 | 1 | 0 | 0 | 1 | 1 | 13 | 22 | 0,59 |  |
| Stansfield et al. (2017) |  | 2 | 2 | 2 | 2 | N/A | N/A | N/A | 2 | 1 | 2 | 0 | 2 | 2 | 2 | 19 | 22 | 0,86 |  |
| Sui et al. (2021) |  | 2 | 2 | 2 | 2 | N/A | N/A | N/A | 2 | 1 | 2 | 2 | 0 | 2 | 2 | 19 | 22 | 0,86 |  |
| Visser, Coetzee, and Claassen (2016) |  | 2 | 2 | 1 | 2 | N/A | N/A | N/A | 2 | 1 | 2 | 2 | 0 | 2 | 2 | 18 | 22 | 0,82 |  |
| Wado et al. (2022) |  | 2 | 2 | 1 | 2 | N/A | N/A | N/A | 2 | 1 | 2 | 0 | 2 | 2 | 2 | 18 | 22 | 0,82 |  |
|  |  | **67** | **57** | **52** | **66** | **0** | **0** | **0** | **67** | **34** | **64** | **46** | **10** | **67** | **64** | 594 |  |  |  |
